# Notching is less, if femoral component sagittal positioning is planned perpendicular to distal femur anterior cortex axis, in navigated TKA

**DOI:** 10.1186/s43019-021-00129-9

**Published:** 2021-12-24

**Authors:** Raj Kanna, Chandramohan Ravichandran, Gautam M. Shetty

**Affiliations:** 1 Department of Orthopaedic Surgery, Prashanth Super Speciality Hospital, Velachery Main Road, Chennai, 600042 India; 2 Knee & Orthopaedic Clinic, Mumbai, India; 3 AIMD Research, Mumbai, India

**Keywords:** Anterior femoral bowing, Computer-assisted knee replacement, Femoral component positioning, Sagittal alignment in TKA, Anterior femoral notching

## Abstract

**Purpose:**

In navigated TKA, the risk of notching is high if femoral component sagittal positioning is planned perpendicular to the sagittal mechanical axis of femur (SMX). We intended to determine if, by opting to place the femoral component perpendicular to distal femur anterior cortex axis (DCX), notching can be reduced in navigated TKA.

**Methods:**

We studied 171 patients who underwent simultaneous bilateral computer-assisted TKA. Femoral component sagittal positioning was planned perpendicular to SMX in one knee (Femur Anterior Bowing Registration Disabled, i.e. FBRD group) and perpendicular to DCX in the opposite knee (Femur Anterior Bowing Registration Enabled, i.e. FBRE group). Incidence and depth of notching were recorded in both groups. For FBRE knees, distal anterior cortex angle (DCA), which is the angle between SMX and DCX, was calculated by the computer.

**Results:**

Incidence and mean depth of notching was less (*p* = 0.0007 and 0.009) in FBRE versus FBRD group, i.e. 7% versus 19.9% and 0.98 mm versus 1.53 mm, respectively. Notching was very high (61.8%) in FBRD limbs when the anterior bowing was severe (DCA > 3°) in the contralateral (FBRE) limbs.

**Conclusion:**

Notching was less when femoral component sagittal positioning was planned perpendicular to DCX, in navigated TKA.

***Level of evidence*:**

Therapeutic level II.

## Introduction

**Fig. 1:**
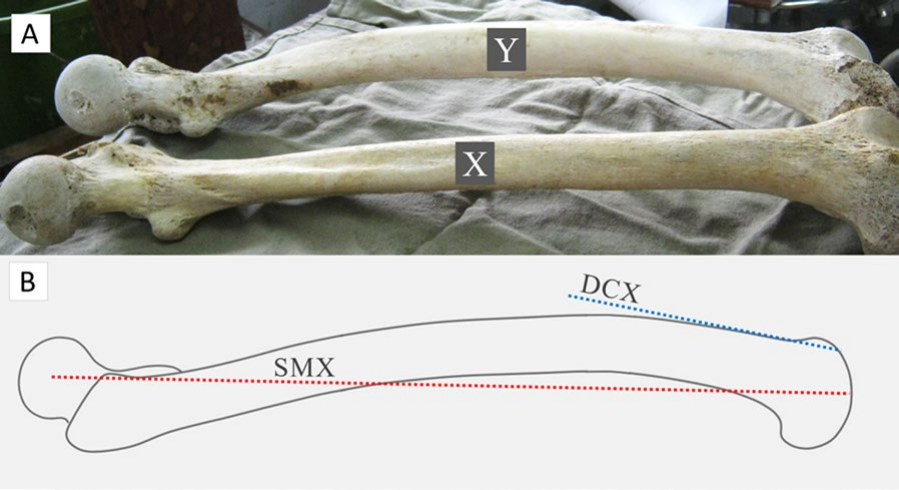
Illustration of varying degrees of anterior bowing in human femur. Femur specimen “X” showing negligible anterior bowing and specimen “Y” showing significant anterior bowing (**a**). Red dotted line represents sagittal mechanical axis of femur (SMX), and the blue dotted line represents distal femur anterior cortex axis (DCX) (**b**)

Human femur has different degrees of anterior bowing [1] (Fig. 1a), which implies that the distal femur anterior cortex axis (DCX) is more flexed than the sagittal mechanical axis of femur (SMX) (Fig. 1b). Studies show that the angle between the DCX and SMX varies widely [2–4], and this can influence the sagittal placement of the femoral component in total knee arthroplasty (TKA) [5]. Further, in navigated TKA, the optimal sagittal alignment of the femoral component is still unknown [6], and most surgeons to date plan to align it either perpendicular [7–9] or in slight (3–5°) flexion [10, 11] to SMX. However, studies show that the risk of anterior femoral notching is high if the femoral component positioning is planned perpendicular to the SMX in navigated TKA [6, 12].

In conventional TKA, the intramedullary rod deviates anteriorly to the SMX in the presence of anterior femoral bowing and the femoral component ends up in a more flexed position [5, 12], which reduces the risk of notching [5]. Hence, we hypothesised that, by opting to place the femoral component perpendicular to the DCX, the risk of notching can be reduced in navigated TKA. The present study is done to compare the incidence and depth of notching between knees in patients undergoing simultaneous bilateral navigated TKA, where femoral component sagittal positioning was planned perpendicular to DCX in one knee and perpendicular to SMX in the opposite knee.

## Materials and methods

### Study design and participants

We prospectively studied 200 patients who underwent same-day bilateral computer-assisted TKA between March 2015 and February 2019. We excluded three patients with previous femur fracture, four who had previous knee surgery, six with inflammatory disease, four with severe liver or kidney problem, two who used steroids, four with femoral stem extenders and six more who failed to follow up. The inclusion criteria were patients who underwent primary, cruciate-substituting, computer-assisted TKAs for primary osteoarthritis of both knees. Our institutional review board approved the study, and informed consent was obtained from all patients.

### Surgical technique and sagittal positioning

All TKAs were performed by a single surgeon (R.K.) using the Kick computer navigation system with its software (Knee 2.6.0, Brainlab, Germany). All patients underwent simultaneous bilateral navigated TKA. A standard medial parapatellar approach was used in all cases. Femoral component sagittal positioning was planned perpendicular to DCX using the femur anterior bowing registration enabled (FBRE) setting in one knee and, perpendicular to SMX, using the femur anterior bowing registration disabled (FBRD) setting in the opposite knee. Randomisation was done using a sealed study number envelope, which was opened before the skin incision was made, and it was blinded to the patients.

Registration was done as per manufacturer’s recommendation. Tibial and femoral arrays were mounted on Schanz pins inserted into the proximal tibia and distal femur. On the femoral side, the following registration steps were common for the knees navigated with FBRE setting (FBRE group) and FBRD setting (FBRD group). First, the computer registered the SMX based on the acquisition of its proximal and distal points, which were centre of the femoral head (acquired by pivoting the femur) and a point 1 cm anterior to the superior border of the intercondylar notch, respectively. Then, the medial and lateral epicondylar points were acquired, and the femoral anterior sizing point, which represents the level of anterior femoral resection, was registered by placing the tip of the pointer on the lateral side of the distal femur anterior cortex, just proximal to the proximal limit of trochlea. Subsequently, the acquisition of the Whiteside’s line was done by holding the pointer along this line, and the surface of the femoral condyles was painted using the pointer, to acquire their most distal and most posterior points, which allowed the software to accurately calculate the distal femoral resection level and the femoral component size. Points were also acquired on the anterior aspect of distal femur to further define the bone model.

**Fig. 2:**
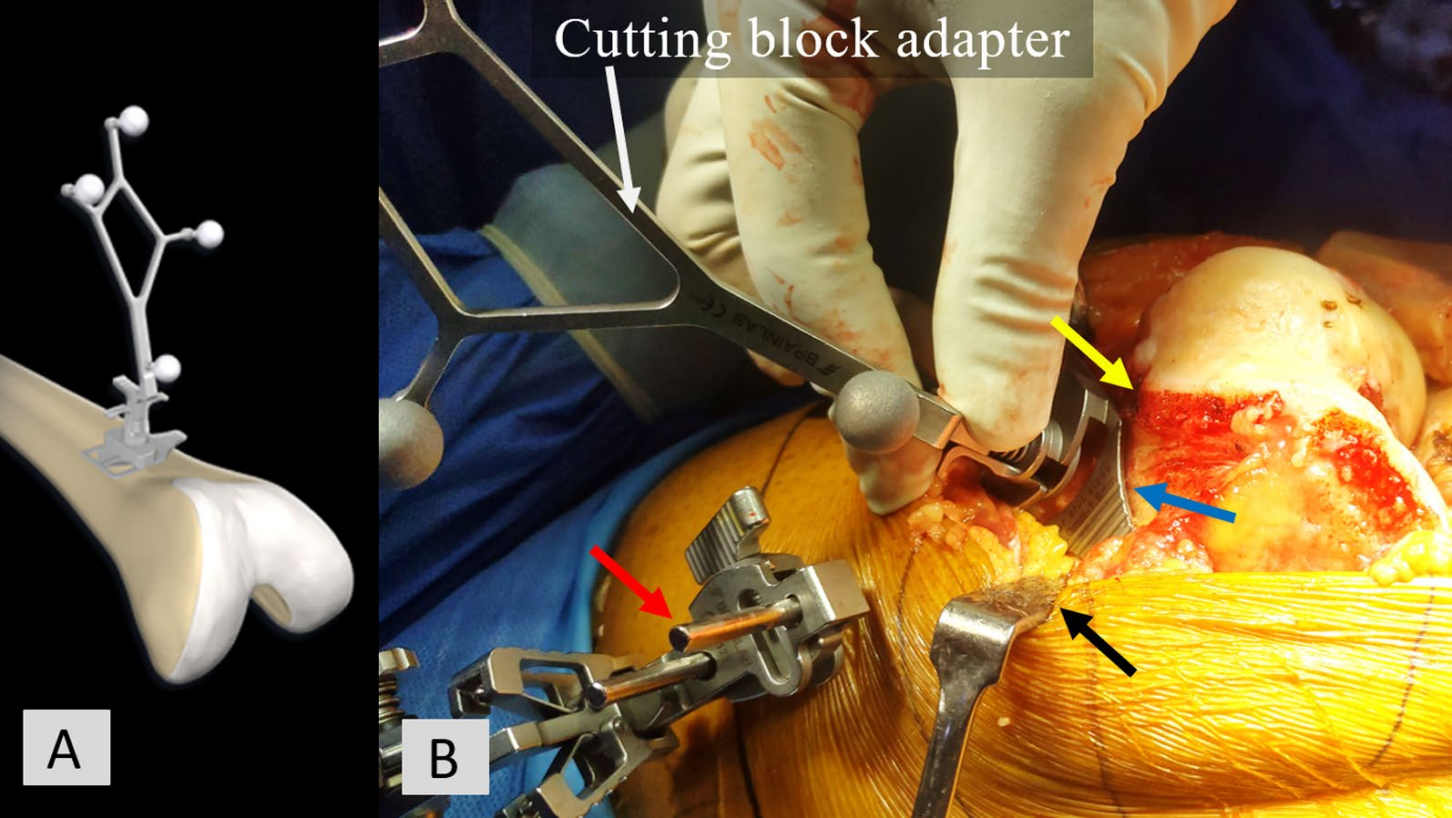
For the knees in the FBRE group, the cutting block adapter was placed over the distal femur anterior cortex to register the DCX (**a**, **b**). Strategies to avoid pitfalls during DCX registration: insertion of the Schanz pins into the distal femur should be proximal enough (red arrow), and trochlear osteophytes should be removed completely (yellow arrow); intervening soft tissue between the cutting block adapter and the distal femur anterior cortex should be avoided (blue arrow); and retraction of the soft tissue flaps should be adequate (black arrow) (**b**)

In addition, for the knees in the FBRE group, the cutting block adapter was placed over the distal femur anterior cortex for a few seconds until the computer registered the DCX (Fig. 2a, b). Common pitfalls during DCX registration and the strategies to avoid them are summarised in Table 1 and illustrated in Fig. 2b. For the knees in the FBRD group, this step was skipped, and therefore DCX was not registered.

**Table 1:** Common pitfalls during DCX registration and the strategies to avoid them

Common pitfalls	Strategies to avoid them
Distal femur Schanz pins and/or the osteophytes along the proximal edge of the trochlea might hinder cutting block adapter placement	Pins should be inserted proximally enough, and the osteophytes should be removed completely
Synovial tissue and the fat pad covering the distal femur anterior cortex interferes with cutting block adapter placement	Soft tissue covering the distal anterior cortex should be incised in the midline and reflect either side meticulously using electrocautery and periosteal elevator
Flaps of the surgical wound could impinge on the cutting block adapter, affecting the accuracy of DCX registration	Soft tissue flaps should be retracted adequately

*DCX* distal femur anterior cortex axis

On the tibial side, all registration steps were identical for knees in both the FBRE and the FBRD group. The proximal and distal points of the tibial mechanical axis were defined by acquiring the posterior aspect of the ACL insertion point and the software calculation based on the medial and lateral malleoli reference points, respectively. Then, the most medial, lateral, and anterior points of the proximal tibia were acquired, and the anteroposterior axis of the proximal tibia was registered by holding the pointer horizontally along the line that connected the tibial attachment of the PCL and the medial third of the tibial tubercle. Modelling of the tibial plateaus were done by placing the tip of the pointer in the deepest point of the plateaus and moving it spirally outwards. Lastly, points were acquired on the anterior aspect of proximal tibia to further define the bone model.

**Fig. 3:**
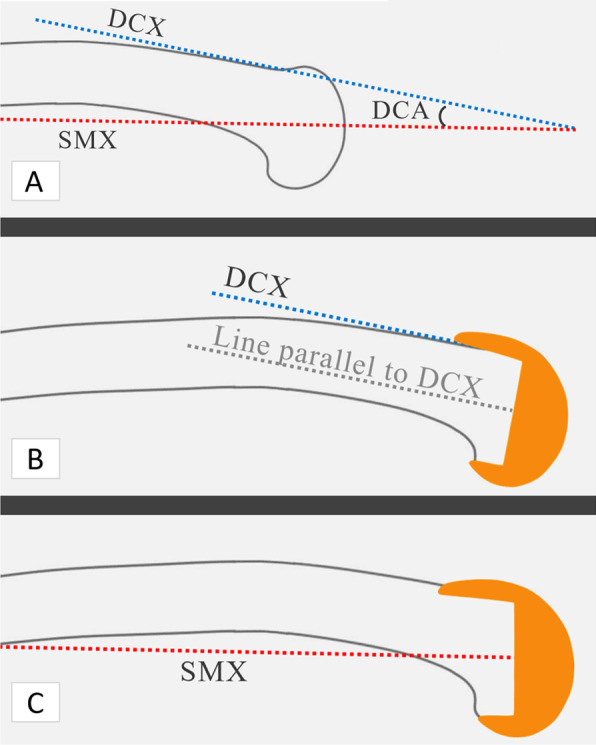
For the knees in the FBRE group, DCA, i.e. the angle between SMX and DCX, was calculated by the computer (**a**). Using navigation, femoral component sagittal positioning was planned perpendicular to the DCX, for the knees in the FBRE group (**b**). Using navigation, femoral component sagittal positioning was planned perpendicular to the SMX, for the knees in FBRD group (**c**)

Distal anterior cortex angle (DCA) is the angle between SMX and DCX, and it essentially quantifies the degree of flexion of the DCX, with respect to the SMX (Fig. 3a). For the knees in the FBRE group, the software calculated the DCA, and using navigation, femoral component sagittal positioning was planned perpendicular to DCX (Fig. 3b) by flexing the femoral component (with respect to the SMX) to the same angle as the calculated DCA. For the knees in FBRD group, DCX was not registered, and therefore DCA values were not calculated and the femoral component sagittal positioning was planned perpendicular to SMX (Fig. 3c).

The rest of the surgical steps were the same in both the FBRE and the FBRD group. Gap balancing technique was used to decide the femoral component rotation, and anterior referencing was used for anteroposterior positioning of the femoral component in both the FBRE and the FBRD group. The P.F.C. Sigma prosthesis (DePuy Orthopaedics, Warsaw, Indiana) was used in both knees of 93 patients, and Attune prosthesis (DePuy Orthopaedics, Warsaw, Indiana) was used in both knees of 78 patients. Surgical technique (including soft tissue release and gap balancing) was identical for both P.F.C. Sigma and Attune knees, expect for the fact that, to perform the anterior femoral cut, we used unslotted and slotted cutting blocks in PFC-Sigma and Attune knees, respectively. Cemented implants were used in all patients, and all had resurfacing of the patella in both knees.

### Hip–knee–ankle (HKA) angle

Standing full-length (hip to ankle) weight-bearing radiographs were obtained in all patients, and the degree of coronal knee deformity or HKA angle was determined before and after surgery.

### Incidence and depth of notching

**Fig. 4:**
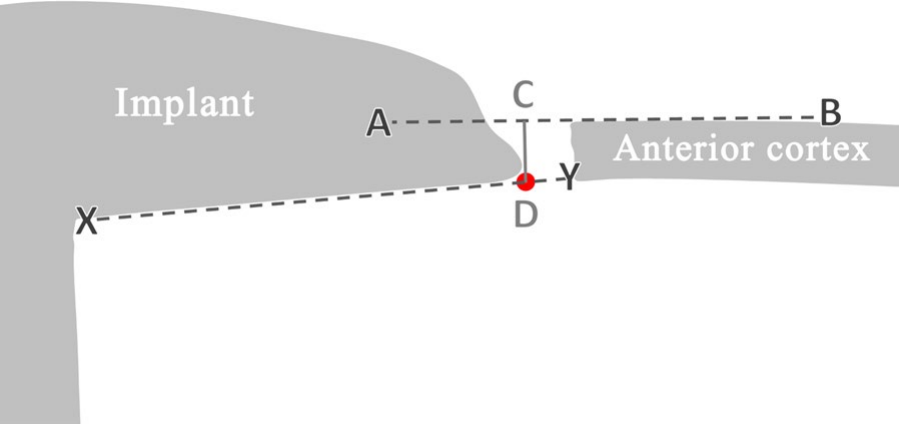
Notch depth (CD) was measured as the perpendicular distance from anterior cortex line (AB) to the point “D” where the anterior resection surface (line *XY*) abutted the implant

**Fig. 5:**
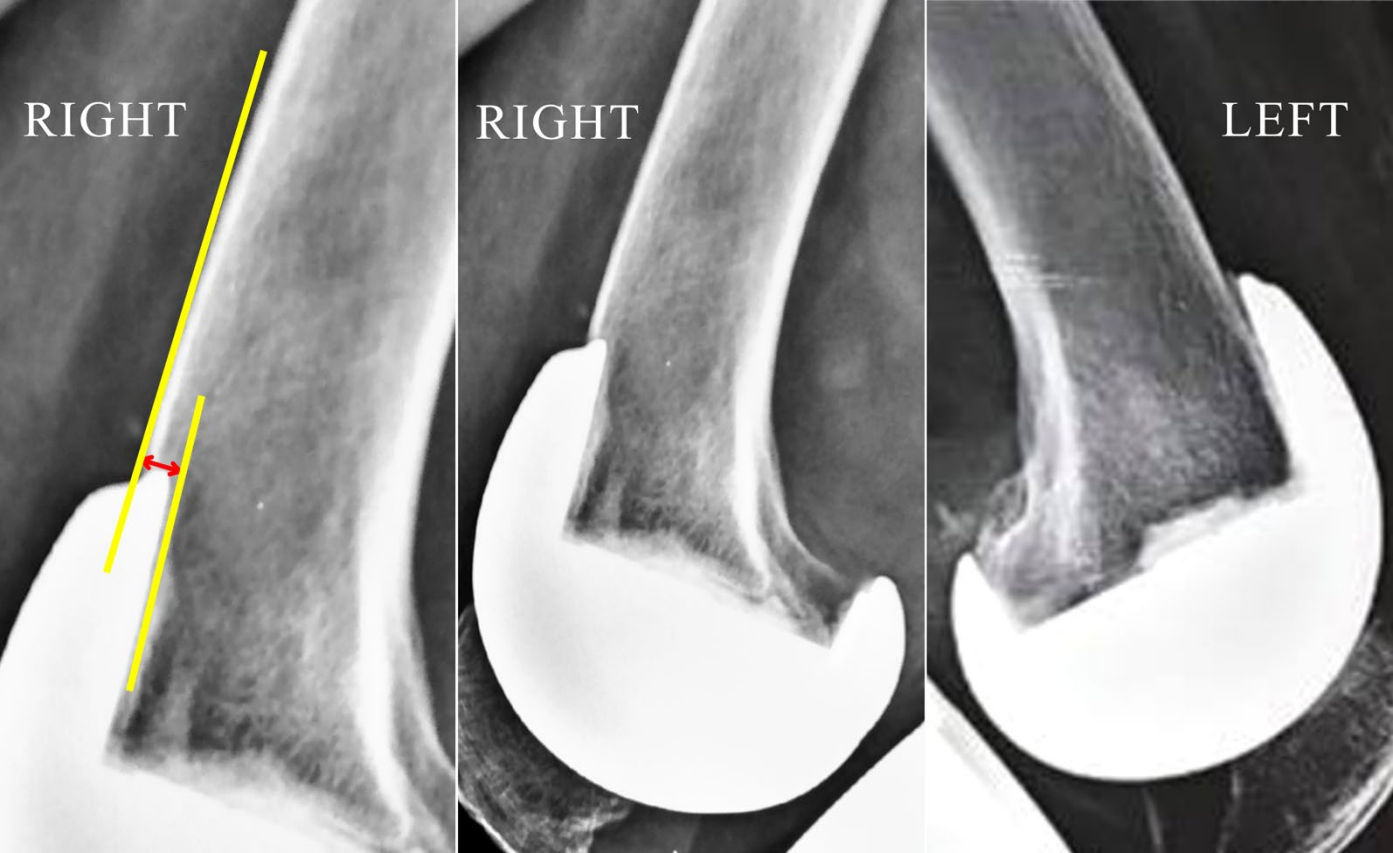
Knee lateral radiographs after simultaneous bilateral navigated TKA in a patient using PFC-Sigma implant. Notching (depth 3.1 mm) seen in right knee (FBRD group), whereas notching was absent in left knee (FBRE group)

**Fig. 6:**
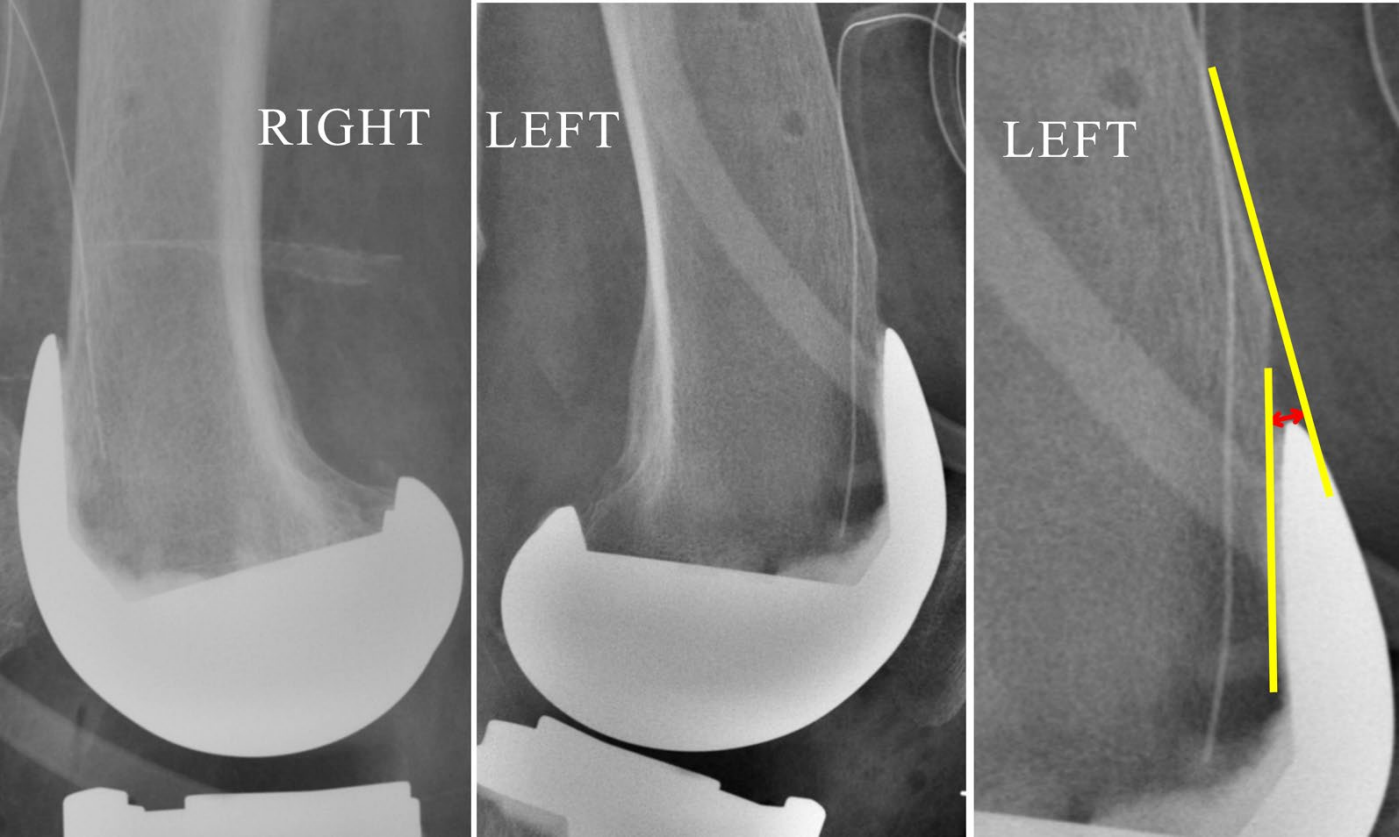
Knee lateral radiographs after simultaneous bilateral navigated TKA in a patient using Attune implant. Notching (depth 2.1 mm) seen in left knee (FBRD group), whereas notching absent in right knee (FBRE group)

Post-surgery lateral knee radiograph was obtained in all patients, and notching, if present, was documented and its depth measured as the perpendicular distance from anterior cortex line to the point where the anterior resection surface abutted the implant (Figs. 4, 5, 6). Notch depth was assessed by a second observer and by the first observer at an interval of minimum 2 weeks from the date of initial assessment, to evaluate inter-observer and intra-observer variability. The following comparisons were done.

- Incidence and depth of notching were compared between FBRD and FBRE groups, in overall patients and in subset of patients with PFC-Sigma and Attune implants.
- Incidence and depth of notching were compared between patients with PFC-Sigma and Attune implants, within the FBRD and FBRE groups.
- Influence of severity of anterior bowing (mild versus severe) on notching was studied in FBRD limbs, where the femoral component sagittal positioning was planned perpendicular to SMX, regardless of the severity of anterior bowing. As the DCA values of FBRD limbs were not available, comparison of incidence and depth of notching within the FBRD limbs was done, based on the DCA values of the contralateral (FBRE) limbs. Our assumption that the DCA values do not differ significantly between the right and left lower limbs was based on the study by Chung et al. [3]. Femur anterior bowing was classified as mild or severe, if the DCA value was ≤ 3° or > 3°, respectively.

### Knee flexion and Knee Society Score (KSS)

Active knee flexion was measured using a goniometer with the patient in supine position. Clinical and functional assessment was done using the KSS (Insall, 1989), which is divided into two sections: a clinical knee score (Knee Society Knee Score, KSKS) and a function score (Knee Society Function Score, KSFS). Knee flexion, KSKS and KSFS were documented before surgery and at two years post-surgery.

### Anterior knee pain (AKP) and femoral component size

At two years post-surgery, patients who had AKP were asked to record pain scores on a visual analogue scale ranging from 0 to 100. The sagittal size of the femoral component initially suggested by navigation and the one chosen finally (after downsizing, because of mediolateral overhang) was noted in all patients.

### Loosening and other complications

Weight-bearing anteroposterior and lateral knee radiographs were obtained in all patients two years post-surgery and were scrutinised for radiolucent lines and signs of loosening. Patients were also scrutinised for complication of notching and navigated TKA, such as periprosthetic fracture, pin site fracture, pin tract infection, surgical site infection, etc., until two years post-surgery. All radiographs were obtained by an experienced technician and uploaded using a computerised imaging system linked to a picture archiving and communication system (PACS). Radiographic images were analysed and measured using Image J image processing and analysis software version 1.41 (National Institutes of Health, Bethesda, MD, USA).

### Statistical analysis

Based on literature [12], the actual number of patients required for our study with the precision/absolute error at 10% and at 95% confidence interval, for a power of 80%, was estimated to be 54. Intra-class correlation estimates and their 95% confident intervals were calculated for intra-observer variability with mean-rating (*k* = 2), two-way mixed-effects model. Spearman’s correlation was used as a measure of inter-rater reliability. Comparison of notch depth, HKA angle, knee flexion, KSKS and KSFS was done using independent *t*-test. Fisher’s exact test was used to compare the incidence of notching and AKP. A *p*-value of < 0.05 was taken to be statistically significant. Data were statistically evaluated with IBM SPSS Statistics for Windows, version 22.0. (IBM Corp., Chicago, IL).

## Results

### Patient demographics

Complete data of 171 patients were available for analysis. Mean age of patients at the time of surgery was 66.5 ± 8.5 years (range 44–89 years). There were 60 (35.1%) male and 111 (64.9%) female patients. Mean body mass index was 29 ± 4 kg/m^2^ (range 21.2–45.4 kg/m^2^).

### Intra-observer and inter-observer variability

There was good test–retest reliability and strong, positive agreement between two observers on notch depth for the knees in FBRD and FBRE groups and the intra-observer and inter-observer agreements were statistically significant (Table 2).

**Table 2:** Intra-observer and inter-observer variability for notch depth

Parameters	Study group	ICC or *r*_s_	95% CI	*p*-Value
Intra-observer variability	FBRD group	ICC = 0.99	0.99–0.99	** < 0.001**
	FBRE group	ICC = 0.98	0.99–0.99	** < 0.001**
Inter-observer variability	FBRD group	*r*_s_ = 0.999	0.79–0.95	** < 0.001**
	FBRE group	r_s_ = 0.998	0.80–0.99	** < 0.001**

*ICC* intra-class correlation coefficient; *r*_s_ Spearman’s correlation; *CI* confidence interval; *FBRD* femur anterior bowing registration disabled; *FBRE* femur anterior bowing registration enabled

*p* < 0.05 is considered statistically significant (bold)

### DCA of FBRE limbs

Out of 171 FBRE limbs, the DCA calculated by the computer was between 0.1–2.0°, 2.1–4.0°, 4.1–6.0° and 6.1–8.0° in 65 (38%), 47 (27.5%), 17 (9.9%) and 1(0.6%) limbs respectively, and the mean DCA was 2 ± 1.7° (range 0–7°).

### Incidence and depth of notching

Comparison of incidence and depth of notching between the FBRD and FBRE groups, in overall patients and in subset of patients with PFC-Sigma and Attune implants, are summarised in Table 3. Notch depth of > 3 mm occurred in 1.17% (2/171) knees in the FBRD group and in none of the 171 knees in the FBRE group. The incidence and mean depth of notching were significantly higher in FBRD limbs when the contralateral (FBRE) limbs had severe anterior bowing, i.e. DCA > 3° (Table 4).

**Table 3:** Comparison of incidence and depth of notching between FBRD and FBRE groups, in overall patients and in subset of patients with PFC-Sigma and Attune implants

Parameters	FBRD group	FBRE group	*p*-Value
Overall patients	*n* = 171	*n* = 171	
Incidence of notching	34/171 (19.9%)	12/171 (7%)	**0.0007**
Mean notch depth (mm)	1.53 ± 0.71 (*R* = 0.2–3.3)	0.98 ± 0.53 (*R* = 0.3–2.2)	**0.009**
Patients with PFC-Sigma implant	*n* = 93	*n* = 93	
Incidence of notching	24/93 (25.8%)	10/93 (10.8%)	**0.0128**
Mean notch depth (mm)	1.58 ± 0.76 (*R* = 0.2–3.3)	1.07 ± 0.53 (*R* = 0.6–2.2)	**0.035**
Patients with Attune implant	*n* = 78	*n* = 78	
Incidence of notching	10/78 (12.8%)	2/78 (2.6%)	**0.0315**
Mean notch depth (mm)	1.41 ± 0.58 (*R* = 0.5–2.2)	0.55 ± 0.35 (*R* = 0.3–0.8)	0.0934

*FBRD* femur anterior bowing registration disabled; *FBRE* femur anterior bowing registration enabled; *mm* millimetre; *n* number of knees; *R* range

*p* < 0.05 is considered statistically significant (bold)

**Table 4:** Comparison of incidence and depth of notching between the limbs of FBRD group, based on the DCA values of the contralateral (FBRE) limbs

Parameters of FBRD limbs	Contralateral (FBRE) limbs with DCA ≤ 3°	Contralateral (FBRE) limbs with DCA > 3°	*p*-Value
Number of limbs	137	34	
Incidence of notching	13/137 (9.5%)	21/34 (61.8%)	** < 0.00001**
Mean notch depth (mm)	1.1 ± 0.56 (*R* = 0.2–2)	1.79 ± 0.67 (*R* = 0.7–3.3)	**0.00293**

*FBRD* femur anterior bowing registration disabled; *FBRE* femur anterior bowing registration enabled; *DCA* distal anterior cortex angle; *mm* millimetre; *R* range

*p* < 0.05 is considered statistically significant (bold)

### HKA angle, knee flexion, KSKS, KSFS and AKP

Comparison of mean HKA angle, knee flexion, KSKS, and KSFS between the FBRE and FBRD groups, both before and after surgery, is summarised in Table 5. Mean knee flexion improved from 128.8 ± 12.9° before surgery to 130.4 ± 10.6° two years after surgery in the FBRD group (*p* = 0.0089) and from 129.3 ± 13.4° before surgery to 133.5° ± 12.2° two years after surgery in the FBRE group (*p* = 0.0037). Mean KSKS improved from 57.7 ± 5.6 before surgery to 90.7 ± 4.7 two years after surgery in the FBRD group (*p* < 0.001) and from 57.3 ± 5.2 before surgery to 91.7 ± 4 two years after surgery in the FBRE group (*p* < 0.001). Similarly, mean KSFS improved from 50.8 ± 5.8 before surgery to 91.7 ± 4.8 two years after surgery (*p* < 0.001) in both the FBRE and the FBRD group. The incidence of AKP was less in the FBRE than in the FBRD group, i.e. 11.1% (19/171) versus 16.4% (28/171), but the difference was not significant (*p* = 0.2086).

**Table 5:** Comparison of the mean HKA angle, knee flexion, KSKS and KSFS between FBRD and FBRE groups, both before and after surgery

Parameters	FBRD group (*n* = 171)	FBRE group (*n* = 171)	*p*-Value
Before surgery			
HKA angle (degrees)	166.6 ± 8.1 (150.3–189.1)	166.7 ± 8.1 (151.4–190.5)	0.8359
Knee flexion (degrees)	128.8 ± 12.9 (100–155)	129.3 ± 13.4 (94–158)	0.7109
KSKS	57.7 ± 5.6 (45–69)	57.3 ± 5.2 (44–68)	0.4967
KSFS	50.8 ± 5.8 (35–60)	50.8 ± 5.8 (35–60)	1
After surgery			
HKA angle (degrees)	179.4 ± 1.7 (175.1–185.6)	179.2 ± 1.7 (175.2–183.6)	0.2336
Knee flexion (degrees)	130.4 ± 10.6 (100–156)	133.5 ± 12.2 (94–158)	**0.0118**
KSKS	90.7 ± 4.7 (80–99)	91.7 ± 4 (79–99)	**0.0328**
KSFS	91.7 ± 4.8 (80–100)	91.7 ± 4.8 (80–100)	1

Range of parameters is shown within brackets

*HKA* hip–knee–ankle; *KSKS* Knee Society Knee Score; *KSFS* Knee Society Function Score; *FBRD* femur anterior bowing registration disabled; *FBRE* femur anterior bowing registration enabled; *n* number of knees

*p* < 0.05 is considered statistically significant (bold)

### PFC-Sigma versus Attune knees

Basic demographics of patients with PFC-Sigma and Attune implants and comparison of means of various parameters between PFC-Sigma and Attune knees within FBRD and FBRE groups, both before and after surgery, are summarised in Table 6.

**Table 6:** Basic demographics of patients with PFC-Sigma and Attune implants and comparison of means of various parameters between PFC-Sigma and Attune knees within FBRD and FBRE groups, both before and after surgery

Parameters	PFC-Sigma implant	Attune implant	*p*-Value
Total number of patients	93	78	
Males	35 (37.6%)	25 (32.1%)	0.5206
Females	58 (62.4%)	53 (67.9%)	
Age (years)	65.8 ± 8.6 (44–83)	67.5 ± 8.5 (50–89)	0.1867
BMI (kg/m^2^)	28.9 ± 3.5 (21.2–40.2)	29.2 ± 4.5 (21.9–45.4)	0.6503
FBRD group (before surgery)	*n* = 93	*n* = 78	
HKA angle (degrees)	167 ± 8.5 (150.8–189.1)	166 ± 7.6 (150.3–182)	0.4473
Knee flexion (degrees)	128.4 ± 13.6 (100–155)	129.2 ± 12 (100–154)	0.6888
KSKS	57.5 ± 5.8 (45–69)	57.9 ± 5.5 (45–68)	0.5822
KSFS	50.2 ± 5.6 (35–60)	51.6 ± 5.9 (40–60)	0.1196
FBRE group (before surgery)	*n* = 93	*n* = 78	
HKA angle (degrees)	167.3 ± 8.2 (153.2–190.5)	165.9 ± 7.8 (151.4–183.8)	0.2669
Knee flexion (degrees)	128.6 ± 13.3 (100–158)	130.2 ± 13.5 (94–155)	0.4288
KSKS	57 ± 5.1 (44–66)	57.6 ± 5.2 (46–68)	0.4378
KSFS	50.2 ± 5.6 (35–60)	51.6 ± 5.9 (40–60)	0.1196
FBRD group (after surgery)	*n* = 93	*n* = 78	
HKA angle (degrees)	179.4 ± 1.9 (175.1–185.6)	179.4 ± 1.5 (176.5–183)	0.8581
Knee flexion (degrees)	128.9 ± 10.9 (100–154)	132.2 ± 10.1 (102–155)	**0.0406**
KSKS	90.3 ± 4.7 (80–99)	91.1 ± 4.7 (82–99)	0.2498
KSFS	91.2 ± 4.8 (80–100)	92.2 ± 4.7 (85–100)	0.1715
Incidence of notching	24 (25.8%)	10 (12.8%)	**0.0364**
Incidence of AKP	19 (20.4%)	9 (11.5%)	0.1475
FBRE group (after surgery)	*n* = 93	*n* = 78	
HKA angle (degrees)	179 ± 1.8 (175.2–183)	179.3 ± 1.7 (176.3–183.6)	0.2038
Knee flexion (degrees)	131.7 ± 11.9 (94–157)	135.7 ± 12.2 (102–158)	**0.0326**
KSKS	91.2 ± 4.4 (79–99)	92.3 ± 3.5 (85–99)	0.0674
KSFS	91.2 ± 4.8 (80–100)	92.2 ± 4.7 (85–100)	0.1715
Incidence of notching	10 (10.8%)	2 (2.7%)	0.0674
Incidence of AKP	13 (14%)	6 (7.7%)	0.228

Range of parameters is shown within brackets

*HKA* hip–knee–ankle; *KSKS* Knee Society Knee Score; *KSFS* Knee Society Function Score; *AKP* anterior knee pain; *FBRD* femur anterior bowing registration disabled; *FBRE* femur anterior bowing registration enabled; *n* number of knees

*p* < 0.05 is considered statistically significant (bold)

### Femoral component sagittal size

In 32/171 (18.7%) patients, the computer suggested one sagittal-size-bigger femoral component for the knees in the FBRD group, compared with that in the FBRE group. Out of these 32 knees in the FBRD group, one sagittal-size-smaller component was used in 15 knees (to avoid mediolateral overhang). A femoral component with same sagittal size, as recommended by the computer, was used in the rest of the 156 knees of the FBRD group and in all 171 knees of the FBRE group. Within the FBRD group, the incidence of notching was not significantly high (*p* = 0.5021) in the 15 knees, where one sagittal-size-smaller femoral component was used, in comparison with that in the rest of the 156 knees, where the same sagittal size component as recommended by the computer was used, i.e. 4/15 (26.7%) versus 30/156 (19.2%).

### Loosening and other complications

**Fig. 7:**
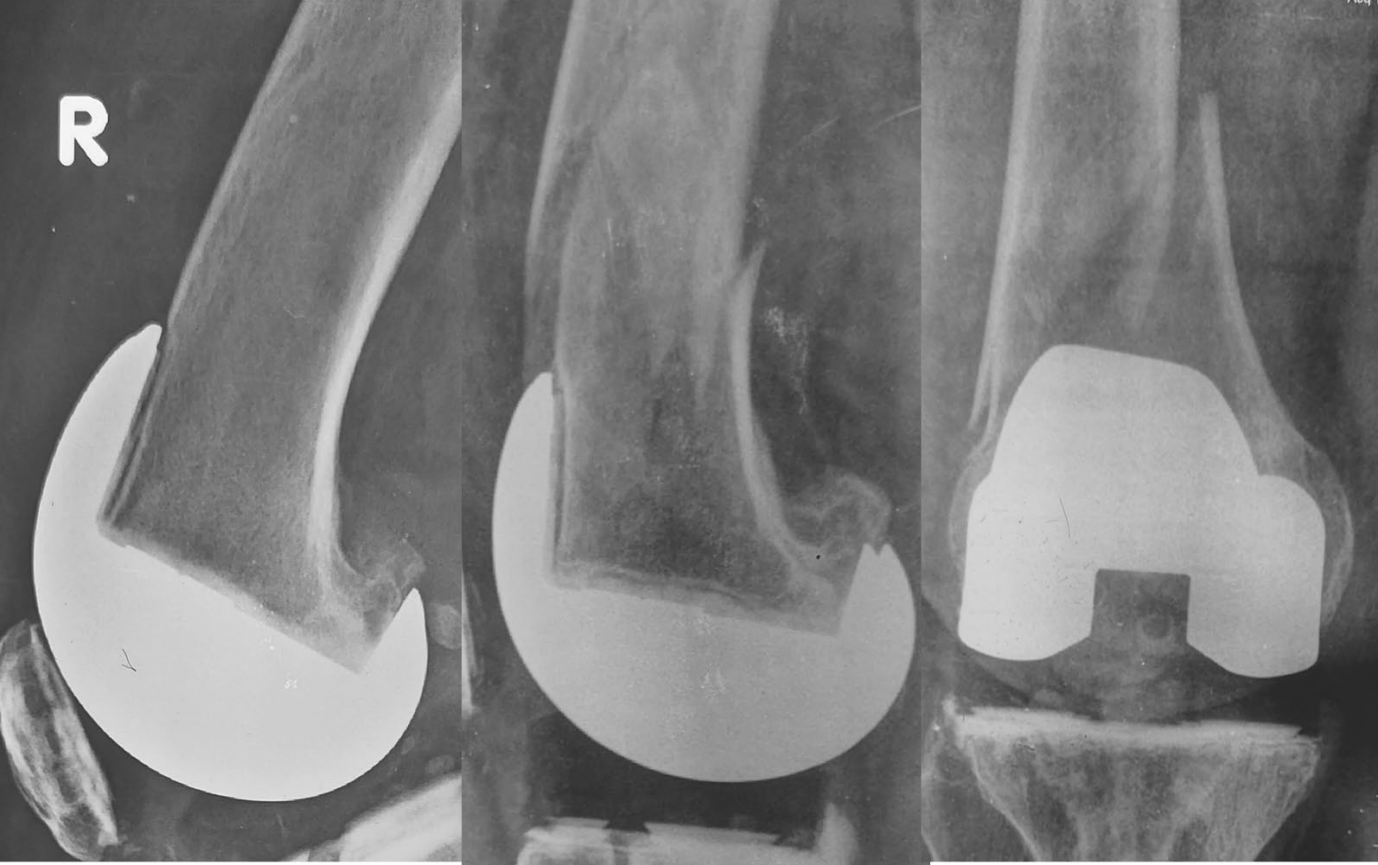
Supra-condylar fracture in a knee with anterior notching, where femoral component sagittal positioning was planned perpendicular to SMX

None of the knees showed progressive radiolucent lines or loosening in the postoperative radiographs at two years post-surgery. Supra-condylar fracture occurred one year post-surgery in one of the knees of the FBRD group which had notching (Fig. 7). The fracture was treated by open reduction and internal fixation, and the patient recovered uneventfully. One patient who developed deep infection 3 weeks after surgery in the FBRE group was treated by debridement and exchange of polyethylene insert and recovered completely. None of the knees had navigation-related complications such as pin tract infection or pin site fracture.

## Discussion

Optimal positioning of the femoral component in the sagittal plane has not yet been defined [13, 14]. Surgeons using navigation, for sagittal positioning of femoral component, continue to make the distal femoral cut perpendicular to SMX [8, 9], although studies have shown that this can increase the risk of notching in navigated TKA [6, 12]. The present study intends to determine, by opting to place the femoral component perpendicular to DCX rather than perpendicular to SMX, if one can reduce the incidence and depth of notching in navigated TKA.

In our study, the incidence of notching was 19.9% in the FBRD group. Similarly, Lee et al. [12] showed that, when the distal femoral cut was made perpendicular to the SMX, the incidence of notching was 16.7% in navigated TKA. In our study, 7% of knees had notching in the FBRE group, and we believe cutting error could be a reason for the notching seen in these knees [15, 16]. Notching was significantly less (*p* = 0.0364) in Attune knees compared with PFC-Sigma knees in the FBRD group, and the difference was nearing significance (*p* = 0.0674) in the FBRE group. We used anterior referencing for both Attune and PFC-Sigma knees, and the anterior flange angle was same (5°) in both implant systems. However, to perform the anterior femoral cut, we used slotted cutting block in Attune and unslotted cutting block in PFC-Sigma knees. Inadvertent lifting of the saw can happen while making the bone cuts in TKA [17] and the propensity to use the saw blade as a lever is high, when an unslotted cutting block is used [18]. This could have increased the cutting error and, therefore, the incidence of notching in PFC-Sigma knees. Ajuied et al. [18] showed that, in the hands of an experienced orthopaedic surgeon, the mean tibial sagittal plane cutting error was significantly high when unslotted versus slotted cutting block was used, i.e. 0.74° ± 0.40° versus 0.67° ± 0.23°, and similarly, on the femoral side, although the difference was not significant, the mean sagittal plane cutting error was high when unslotted versus slotted cutting block was used, i.e. 1.5° ± 0.29° versus 1.2° ± 0.36°. Also, Love et al. [16] showed that deliberate lifting of the saw blade or using unslotted cutting block can cause significant sagittal plane cutting error in TKA.

In our study, mean notch depth was 1.53 ± 0.71 mm for the knees in the FBRD group, while in the study by Lee et al. it was 3.32 ± 1.54 mm [12], where the distal femoral cut was made perpendicular to SMX using navigation. Culp et al. [19] measured notch depth on the lateral radiographs and showed that violation of the anterior cortex up to 3 mm reduces its torsional strength by 29%. In our study, only 1.16% (2/171) knees had notch depth > 3 mm in the FBRD group, whereas in the study by Lee et al. [12] > 3 mm notching was seen in at least 9% (7/78) of their navigated TKAs. While we used anterior referencing, in the latter study [12] the authors used posterior referencing, and this could be the reason for the differences in the results between the two studies.

To the best of our knowledge, the current study is the first to evaluate the influence of severity of anterior bowing on notching in TKA. The incidence and mean depth of notching were significantly higher in FBRD limbs when the contralateral limbs had severe anterior bowing, i.e. DCA > 3° (Table 4). Chung et al. [3] measured the DCA of right and left femurs from their true lateral radiograph in 100 adults, and showed that that there was no significant difference in the mean angles (*p* = 0.675) between the sides, i.e. 2.9° ± 1.9° versus 3.0° ± 1.8°, respectively. Also, in their study, more than 2° difference in DCA between the sides was seen in only 6% of subjects.

Past studies show that surgeons have aimed to position the femoral component within 3° [10] or 5° flexion [11] with respect to the SMX, while using navigation. However, in 19.9% (34/171) of knees the DCA was > 3°, and in 3.5% (6/171) of knees it was > 5°, in the FBRE group in our study. Therefore, in theory, flexing the femoral component up to 3° or 5° with respect to the SMX may still not be adequate to avoid notching in knees with DCA > 3° or > 5°, respectively. The maximum DCA recorded in our study was 7°. Similarly, a maximum DCA of 7.2° and 8.5° was recorded in the studies by Bao et al. [2] and Chung et al. [3], respectively.

In the present study, the mean knee flexion was significantly higher for the knees in the FBRE group compared with that in the FBRD group at two years post-surgery. Further, within FBRD and FBRE groups, the mean knee flexion was significantly high in Attune knees compared with PFC-Sigma knees at two years post-surgery. Antony et al. [20] showed that there was a positive correlation between the femoral component sagittal angle and the knee flexion, and Song et al. [21] in their study of 600 TKAs showed that Attune knees had better range of motion in comparison with PFC-Sigma knees.

In our study, mean KSKS was significantly higher for the knees in the FBRE group compared with that in the FBRD group at two years post-surgery. Further, mean KSKS was higher for Attune knees compared with PFC-Sigma knees in the FBRE group, and this difference was nearing significance (*p* = 0.0674) at two years post-surgery. Similarly, studies have shown that, with flexed femoral component, the quadriceps moment arm increases, resulting in improved KSFS in TKA [22, 23], and different authors [21, 24] have shown favourable clinical results with Attune knees in comparison with those with PFC-Sigma knees.

Although insignificant (*p* = 0.2086), the incidence of AKP was high in the FBRD versus FBRE group, i.e. 16.4% versus 11.1% at two years post-surgery, in the present study. Scott et al. [25] showed that femoral component extension of ≥ 0.5° predicted AKP with 87% sensitivity, and Kang et al. [26] concluded that, in comparison with the extended condition, a neutral-to-mild flexed femoral component decreased the patella–femur contact stress, which may contribute to decreasing the AKP in TKA. Similarly, although insignificant (*p* = 0.1475 and 0.228), the incidence of AKP was less in Attune versus PFC-Sigma knees in both the FBRD and the FBRE group in our study. Similarly, past studies [27, 28] have shown that the incidence of AKP was less in patients with Attune implant in comparison with that in patients with PFC-Sigma implant.

In 32 out of 171 patients, the computer suggested one sagittal-size-bigger femoral component for the knees in the FBRD group, compared with that in the FBRE group in the current study. Nakahara et al. [29] showed that a 3° and a 5° extension of the distal femoral cut increased the sagittal femoral diameters by 2 and 3 mm, respectively, whereas a 3° and a 5° flexion of the distal cut decreased the sagittal diameter by 2 and 3 mm, respectively. Within the FBRD group, the incidence of notching was not significantly higher in the 15 out of total 171 knees, where one sagittal-size-smaller femoral component (as compared with the one recommended by the computer) was used, and this is understandable as we used anterior referencing for anteroposterior positioning of the femoral component in all knees in the present study.

In our opinion, registration of DCX is neither technically demanding nor time consuming (takes a few seconds), and although not within the scope of the current study, we believe that this additional step may have no noticeable impact on the learning curve involved in computer-assisted TKA. While flexed femoral component avoids notching [5] and helps in improving functional outcome [22, 23], excessive flexion of the component can cause tibial post-impingement [26], and this is design dependent [30]. However, when it comes to conventional TKA, Banks et al. [31] showed that neutral placement is biased by an average 10° of hyperextension between femoral and tibial components, secondary to the anterior femoral bowing and posterior tibial slope.

Our study has certain limitations. We used gap balancing technique in which the femoral component can be more externally rotated [32], and this can increase the risk of notching [6]. Further, soft tissue release can influence femoral component rotation in gap balancing technique [33]. Femoral component rotational alignment was not assessed in the present study. However, the mean pre- and post-operative HKA angles were not significantly different between the FBRD and FBRE groups, and we used the same soft tissue release technique in all knees. Therefore, it is unlikely that our technique would have influenced the final outcome of the present study. Although notch depth can be accessed more accurately using 3D computer topography (CT) scan, we used radiographs for these measurements as they are easily available and are cost-effective, not to mention the risk of radiation involved in 3D CT scanning of both knees. Further there was good test–retest reliability and strong agreement between two observers for notch depth in our study.

## Conclusion

The present study shows that, irrespective of the implant used (PFC-Sigma or Attune), by opting to position the femoral component perpendicular to DCX rather than perpendicular to SMX, notching can be reduced in navigated TKA. Surgeons using navigation should be cautious when they suspect significant anterior bowing, as the risk of notching was very high (61.8%) in the FBRD group when the contralateral limbs had severe anterior bowing (DCA > 3°). Further, in such cases, surgeons should consider an implant which allows maximal hyperextension between components, to avoid cam-post impingement. Prosthesis manufacturers should consider future designs which can accommodate more hyperextension, to deal with populations where severe bowing is not uncommon.

## Data Availability

All data generated or analysed during this study are included in this published article.

## Abbreviations

DCX: Distal femur anterior cortex axis
SMX: Sagittal mechanical axis of femur
TKA: Total knee arthroplasty
FBRE: Femur bowing registration enabled
FBRD: Femur bowing registration disabled
DCA: Distal anterior cortex angle
HKA: Hip–knee–ankle
KSS: Knee Society Score
KSKS: Knee Society Knee Score
KSFS: Knee Society Function Score
AKP: Anterior knee pain
PACS: Picture archiving and communication system
